# Impact of meteorological factors on the incidence of bacillary dysentery in Beijing, China: A time series analysis (1970-2012)

**DOI:** 10.1371/journal.pone.0182937

**Published:** 2017-08-10

**Authors:** Long Yan, Hong Wang, Xuan Zhang, Ming-Yue Li, Juan He

**Affiliations:** 1 School of Preclinical Medicine, Beijing University of Chinese Medicine, Beijing, China; 2 Hong Kong Chinese Medicine Clinical Study Centre, School of Chinese Medicine, Hong Kong Baptist University, Hong Kong, China; 3 Department of Applied Language, Peking University Health Science Center, Beijing, China; International Nutrition Inc, UNITED STATES

## Abstract

**Objectives:**

Influence of meteorological variables on the transmission of bacillary dysentery (BD) is under investigated topic and effective forecasting models as public health tool are lacking. This paper aimed to quantify the relationship between meteorological variables and BD cases in Beijing and to establish an effective forecasting model.

**Methods:**

A time series analysis was conducted in the Beijing area based upon monthly data on weather variables (i.e. temperature, rainfall, relative humidity, vapor pressure, and wind speed) and on the number of BD cases during the period 1970–2012. Autoregressive integrated moving average models with explanatory variables (ARIMAX) were built based on the data from 1970 to 2004. Prediction of monthly BD cases from 2005 to 2012 was made using the established models. The prediction accuracy was evaluated by the mean square error (MSE).

**Results:**

Firstly, temperature with 2-month and 7-month lags and rainfall with 12-month lag were found positively correlated with the number of BD cases in Beijing. Secondly, ARIMAX model with covariates of temperature with 7-month lag (β = 0.021, 95% confidence interval(CI): 0.004–0.038) and rainfall with 12-month lag (β = 0.023, 95% CI: 0.009–0.037) displayed the highest prediction accuracy.

**Conclusions:**

The ARIMAX model developed in this study showed an accurate goodness of fit and precise prediction accuracy in the short term, which would be beneficial for government departments to take early public health measures to prevent and control possible BD popularity.

## Introduction

Bacillary dysentery (BD) is an enteric infection caused by *Shigella* bacteria that leads to a spectrum of clinical presentations such as fever, abdominal discomfort, tenesmus, and diarrhea with stool containing pus and blood[1, 2]. Severe cases may suffer from systemic poisoning and other life-threatening complications. BD is generally transmitted through faecal-oral route via contaminated food and water. BD occurs more frequently in developing countries with poor sanitation and unsafe water supply, and most cases are seen in summer and autumn, which indicates the role meteorological factors might play in BD incidence.

Shigellosis, as a major global health problem, poses threats to both developed and developing countries[3], with no exception for China. Despite the fact that China has witnessed a substantial drop of BD morbidity and mortality in the 1990s owing to improved availability to health care service and antibiotics supply, BD still has a relatively high incidence, especially among certain disadvantaged groups including children, senior citizens, and people of low socioeconomic standing[4].

Some studies concluded that the transmission of infectious diseases including BD might be influenced by meteorological factors[5–9]. For example, studies in Peru[10], Pacific Islands[11] and China[2, 12–16] have investigated how BD incidence was related to the climatic conditions. According to a study in Jinan city[12], maximum temperature, minimum temperature, rainfall, relative humidity, and air pressure were all significantly correlated with the number of dysentery cases. This study further concluded that a rise of 1°C in maximum temperature might be associated with more than 10% increase in BD cases. Another report on Harbin and Quzhou city demonstrated that the mean water vapor pressure over the previous months might result in higher relative risk of BD transmission[2]. Furthermore, a study in Wuhan city reported that mean temperature (excess risk for 1°C increase being 0.94% on lag day 2) exhibited a positive correlation with BD while negative correlation was found in terms of relative humidity (excess risk for 1% increase being -0.21%) and rainfall (excess risk for 1 mm increase being -0.23%)[15]. However, how meteorological variables act on the transmission of BD is still far from clear with very few studies quantifying the relationship between BD and meteorological factors.

Similar studies on such relationship set in Beijing have also been documented, but limitations of the previous researches cannot be overlooked. In some studies, although correlation coefficient was computed through regression analysis, the autoregressive relationship of BD cases and weather series has not been taken into account[17]. Other studies merely analyzed time series without enough notice of hysteresis[18]. Therefore, a time series analysis was conducted in this study, aiming at exploring and, if possible, quantifying the relationship between meteorological variables and BD incidence in Beijing city based on data from 1970 to 2012 on monthly BD cases and meteorological factors (including temperature, rainfall, relative humidity, vapor pressure, and wind speed). The ARIMAX models were established to fit and predict the incidence of BD taking into consideration lagged effects and autocorrelation of BD cases.

## Materials and methods

### Study location

Beijing, capital of China, is located in the northern part of the vast North China Plain with the north latitude 39°56’ and east longitude 116°20’. The prevailing northern temperate semi-humid continental monsoon climate brings Beijing distinct seasons, hot rainy summer, cold dry winter, and relatively short spring and autumn. The past decades have witnessed an enormous population growth from 7,712,000 in 1970 to 20,693,000 in 2012. (http://www.bjstats.gov.cn/tjsj/cysj/201511/t20151109_311727.html).

### Data collection

#### Data of BD

Time series data on monthly BD cases from January 1970 to December 2012 were obtained from Beijing Center for Disease Control and Prevention. The BD cases were diagnosed according to the criterion from China’s former Ministry of Health (currently National Health and Family Planning Commission of the PRC). The disease surveillance data used in this study were collected from the report of legal infectious disease of Beijing City.

#### Meteorological data

Original daily data of five meteorological elements (including temperature, rainfall, relative humidity, vapor pressure, and wind speed) from 1970 to 2012 were provided by Beijing Meteorological Bureau, and average monthly values were calculated based thereupon.

### Statistical analysis

The well-known autoregressive integrated moving average (ARIMA) model for time series analysis is widely used to describe and predict epidemic prevalence for its accuracy and practicality[7, 19–21]. Developed from the ARIMA model, the multiplicative seasonal ARIMA model that incorporates seasonal period performs better in the presence of an obvious seasonal pattern. An ARIMA(*p*,*d*,*q*)(*P*,*D*,*Q*)_*s*_ model comprises 7 parameters: *p* and *P* are the orders of general and seasonal auto regeression(AR) respectively; *q* and *Q* are the general and seasonal moving average(MA) orders respectively; *d* and *D* are the numbers of general and seasonal differencing respectively; *s* denotes periodicity. It can be delineated as follows[20]:

$$\Phi (B)\emptyset (B){\left(1-B\right)}^{d}{{\left(1-B^{s}\right)}^{D}Z}_{t}=\Theta (B)\theta (B)\varepsilon_{t}$$ (1)

with *Z*_*t*_ representing the value of time series at time *t*, and *ε*_*t*_ a white noise series. *B* here refers to a backward shift operator (e.g. *BZ*_*t*_ = *Z*_*t*−1_). ∅(*B*) = 1 − ∅_1_*B* − ⋯ ∅_*p*_*B*^*p*^ and Φ(*B*) = 1 – Φ_1_*B*^*s*^ − ⋯Φ_*P*_*B*^*Ps*^ denote the general and seasonal auto-regressive operators respectively. θ(*B*) = 1 − θ_1_*B* − ⋯θ_*q*_*B*^*q*^ and Θ(*B*) = 1 − Θ_1_*B*^*s*^ − ⋯Θ_*Q*_*B*^*Qs*^ stand for the general and seasonal moving average operators respectively.

The ARIMAX model extends the capability of the ARIMA model in that it integrates into time series modeling external information such as temperature, vapor pressure, and other meteorological factors. An ARIMAX model can be expressed as follows:

$$Y_{t}=\sum_{i=m_{1}}^{m_{2}}\beta_{i}X_{t-i}+Z_{t}$$ (2)

In this model, *Y*_*t*_ represents the time series of the response variable and *X*_*t*_ is a covariate time series that, hopefully, could help explain or forecast *Y*_*t*_. *Z*_*t*_ satisfies Eq (1) and *β*_*i*_ refers to the coefficient. The expressions *m*_1_ and *m*_2_ are the lower and upper limits of the lag respectively.

The ARIMAX model was built to evaluate the relationship between monthly BD cases and weather factors, and then to forecast the BD cases. The steps of the whole process were listed as below.

1. First, the ARIMA model was applied to the time series of BD cases from 1970 to 2004. Auto correlation function (ACF) and partial auto correlation function (PACF) plots guided identification of the model structure.
2. Second, which covariate at which lag shall enter into the model was determined through inspecting the sample cross-correlation function (CCF) on the basis of the prewhitened data. Since it was difficult to assess the dependence between the two processes with strongly autocorrelation, prewhitening was conducted to disentangle the linear association from their autocorrelation. More importantly, the theoretical cross-correlation of the prewhitened processes at lag *i* was proportional to the regression coefficient *β*_*i*_ [20].
3. Third, meteorological factors selected through (b) were incorporated as covariates into the ARIMAX model. As to the inclusion standard of meteorological factors, only those with statistically significant regression coefficients and the ability of lowering the Akaike Information Criterion (AIC) value would eventually be incorporated into the model. For an infinite-order ARMA process, minimizing AIC value would lead to an optimal ARMA model that was closest to the actual process among the class of models under study[20].
4. Finally, the ARIMAX models were used to predict the BD cases from 2005 to 2012. The prediction accuracy was evaluated by the mean square error (MSE).

The maximum likelihood method was adopted for estimation of the parameters. The Ljung-Box Q test was performed to determine whether the residual series were white noise. The whole process of data analysis was carried on in R 3.3.1.

## Results

### Description and univariate analysis

**Fig 1:**
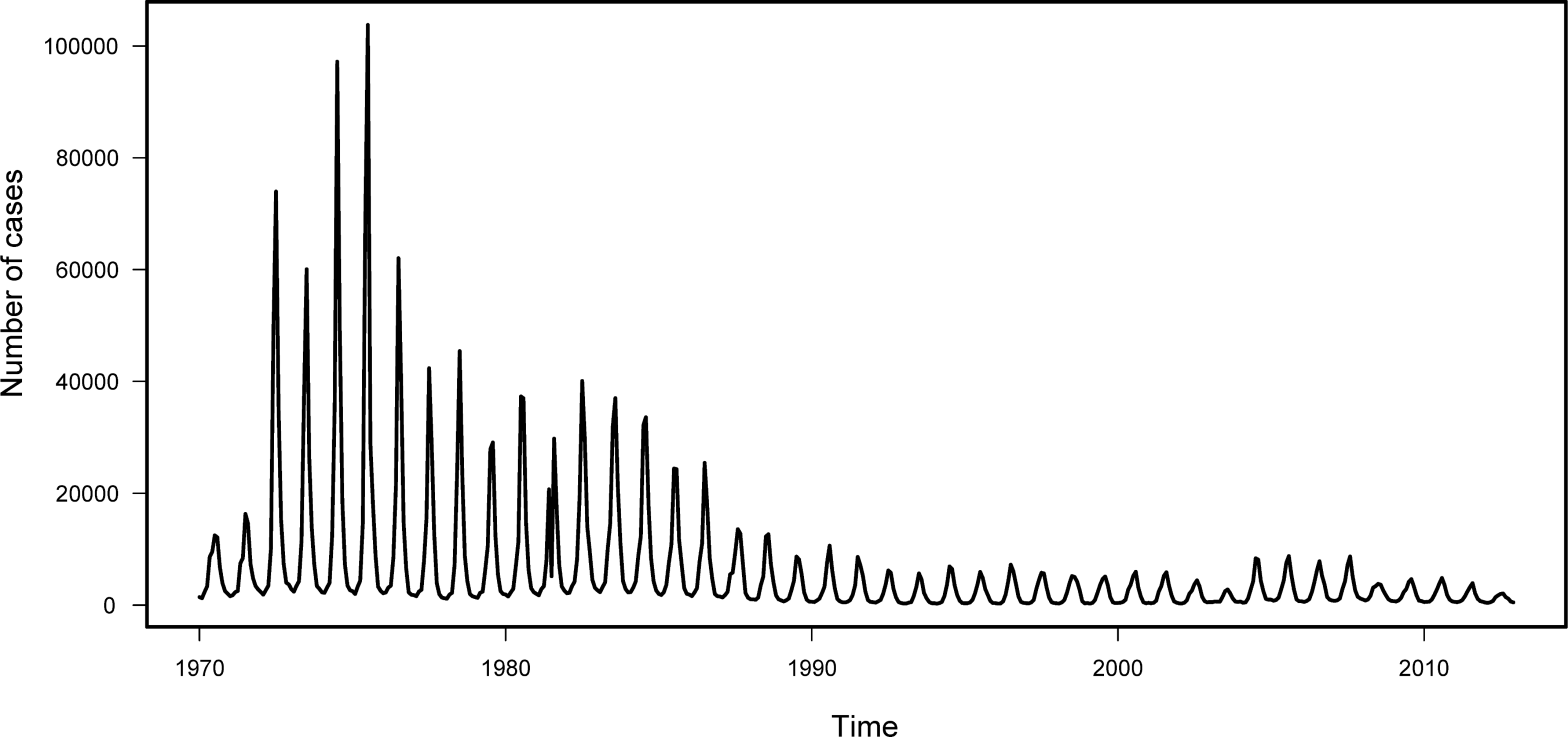
The monthly number of BD cases in Beijing, 1970–2012.

Altogether, 3,148,389 BD cases were reported in Beijing from 1970 to 2012 (Fig 1). In 43 years’ fluctuation, BD cases peaked in July of 1975 with 103,774 counts, and the second highest peak appeared in July of 1974 with 97,203 cases. Besides these major epidemic peaks, other periods with the highest BD incidence rates also arose before 1986, after which a considerable decline was noticed, lasting until 2012. A distinct seasonal distribution was also observed in the figure, since most of the BD cases occurred during summer and autumn, roughly from May to September.

### The analysis of the ARIMA model

**Fig 2:**
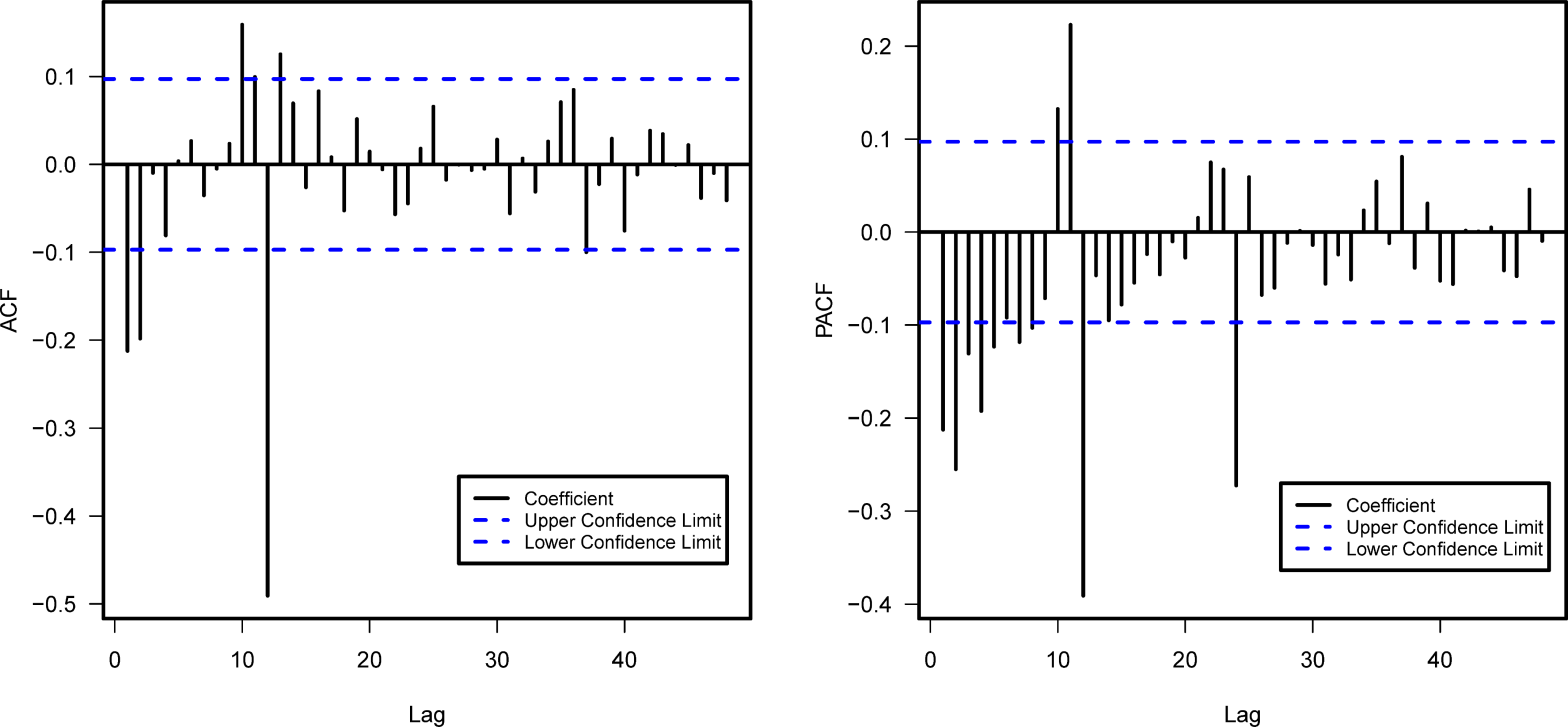
Results of ACF and PACF for time series analysis using 1-step non-seasonal and 1-step seasonal differences.

ARIMA model was built on the time series of BD cases from 1970 to 2004. To stabilize fluctuation of time series data, logarithmic transformation was applied to the time series of BD cases. As the time series plot of the logarithms of BD cases showed a downward trend along with an obvious seasonal distribution, first order non-seasonal and seasonal differences were applied simultaneously. Then, the graphs for ACF and PACF were used as guides for identifying the model structure (Fig 2). Notice that the sample ACF values exceeded the critical value at lag 12, and that the sample PACF values exceeded the critical values at lag 24, the upper limits of the seasonal parameter *Q* and of the seasonal parameter *P* were 1 and 2 respectively. Because the non-seasonal part of ACF cuts off at lag 2, the maximum value of the non-seasonal parameter *q* was 2. As for the non-seasonal parameter *p*, the maximum value thereof was assumed to be 2. In order to search out the most suitable model, all 54 ARIMA models under the following four conditions were carried out: *p* = 0, 1 or 2; *q* = 0, 1 or 2; *P* = 0, 1 or 2; *Q* = 0 or 1. Table 1 lists the five ARIMA models with the lowest AIC value. Although the AIC values of ARIMA(1,1,2)(2,1,1)_12_ and ARIMA(2,1,1)(2,1,1)_12_ were lower than that of ARIMA(1,1,1)(2,1,1)_12_, the estimation of the 2-order moving average coefficient θ_2_ of ARIMA(1,1,2)(2,1,1)_12_ and that of the 2-order autoregressive coefficient ∅_2_ of ARIMA(2,1,1)(2,1,1)_12_ were not statistically significant (θ_2_ = −0.170, p = 0.063; ∅_2_ = −0.100, p = 0.069). As was shown in Table 2, the estimation of all autoregressive and moving average coefficients of ARIMA(1,1,1)(2,1,1)_12_ model were significantly nonzero. The *p*-value of Ljung-Box Q test for this model was 0.271, which indicated that the model captured the dependence between variables in the time series. In summary, the ARIMA(1,1,1)(2,1,1)_12_ model was by far the most suitable one. (Details were summarized in S1 File on the analysis of ARIMA model.)

**Table 1:** Comparison of ARIMA models.

Model	AIC	Ljung-Box test		
		Statistic	DF	Significance
**ARIMA(1,1,2)(2,1,1)_12_**	11.086	6.079	6	0.414
**ARIMA(2,1,1)(2,1,1)_12_**	11.155	6.287	6	0.392
**ARIMA(1,1,1)(2,1,1)_12_**	12.408	8.758	7	0.271
**ARIMA(2,1,2)(2,1,1)_12_**	12.761	5.518	5	0.356
**ARIMA(2,1,2)(1,1,1)_12_**	14.834	5.712	6	0.456

**Table 2:** Parameters in ARIMA(1,1,1)(2,1,1)_12_ model.

Coefficient	Estimate	Standard Error	t	*p*-value
**∅_1_**	0.532	0.057	9.340	< 0.001
**θ_1_**	-0.896	0.029	-30.990	< 0.001
**Φ_1_**	0.234	0.069	3.391	< 0.001
**Φ_2_**	0.210	0.068	3.070	0.002
**Θ_1_**	-0.947	0.058	-16.418	< 0.001

∅_1_**:** 1-order auto-regressive coefficient, θ_1_**:** 1-order moving average coefficient, Φ_1_ and Φ_2_**:** 1-order and 2-order seasonal auto-regressive coefficients, Θ_1_**:** 1-order seasonal moving average coefficient

### Meteorological factors selection

**Fig 3:**
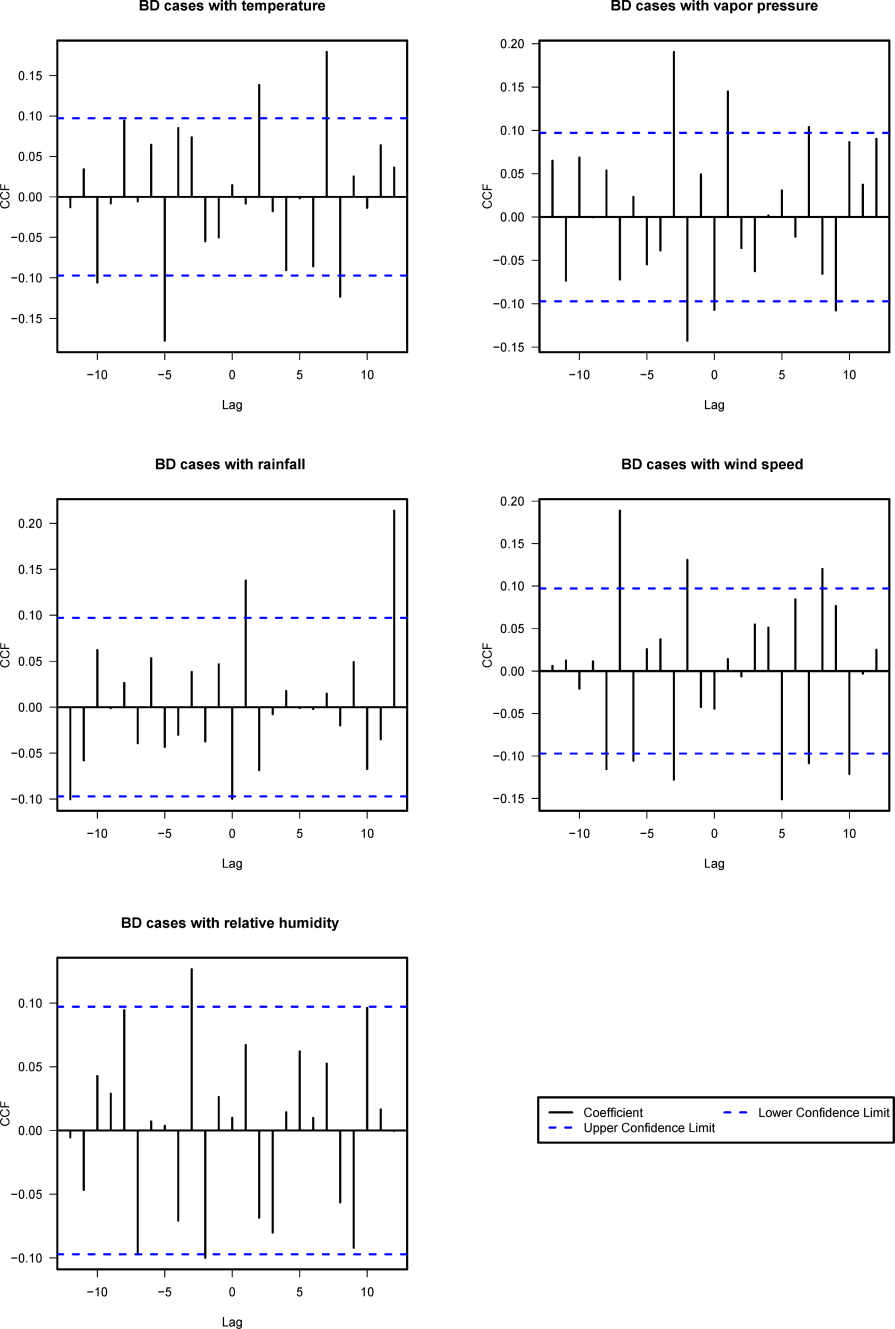
Cross-correlation between prewhitened logarithmic BD cases and weather variables.

CCF was adopted as a tool to explore the relationship between meteorological factors and BD cases. Data on monthly BD cases and monthly meteorological factors were prewhitened by the fitted ARIMA model in the previous section. Fig 3 showed the cross-correlation between the prewhitened weather variables (monthly temperature, rainfall, relative humidity, vapor pressure, and wind speed) and BD cases at lags of 0 to 12 months. Only positive lags would be considered because the positive value indicated that meteorological factors could affect BD counts a certain period of time later. Except relative humidity, the rest four factors proved to be statistically significantly associated with BD. For example, the CCF between temperature and BD cases was significant at lags 2, 7 and 8, suggesting the presence of relationship between temperature at these three lags and BD counts. All the relationship found through CCF were to be used in establishing the ARIMAX model.

### Establishing the ARIMAX model

Efforts have been made on incorporating the above four climatic factors as covariates into the ARIMAX model. First, the model was tested with single lagged meteorological factor. As was shown in Table 3, only temperature at lags of 2 and 7 and rainfall at lag 12 had statistically significant parameters and could further lower the AIC value. Further analysis manifested that meteorological factors in order of importance were rainfall (at lag 12), temperature (at lag 7) and temperature (at lag 2). Lower AIC value endowed the factor with more importance. Afterwards, these meteorological factors were used to build the ARIMAX model with multiple independent variables. Four ARIMAX models with multiple independent variables lowered the AIC value, and the ARIMAX model with covariates of temperature at lags 2, 7 and rainfall at lag 12 reached the lowest AIC value.

**Table 3:** ARIMAX models with different meteorological factors.

Model	Meteorological factors						AIC
	Variables	Lag	Estimate	Standard Error	t	*p*-value	
**ARIMA(1,1,1)(2,1,1)_12_**	-	-	-	-	-	-	12.408
**ARIMAX(1,1,1)(2,1,1)_12_**	T	2	0.018	0.009	2.071	0.039*	10.223**
**ARIMAX(1,1,1)(2,1,1)_12_**	T	7	0.019	0.009	2.256	0.025*	9.384**
**ARIMAX(1,1,1)(2,1,1)_12_**	T	8	-0.008	0.009	-0.942	0.347	13.528
**ARIMAX(1,1,1)(2,1,1)_12_**	R	0	-0.008	0.008	-1.053	0.293	13.292
**ARIMAX(1,1,1)(2,1,1)_12_**	R	1	0.009	0.008	1.160	0.247	13.051
**ARIMAX(1,1,1)(2,1,1)_12_**	R	12	0.022	0.007	3.000	0.003*	5.616**
**ARIMAX(1,1,1)(2,1,1)_12_**	V	0	-0.011	0.010	-1.105	0.270	13.196
**ARIMAX(1,1,1)(2,1,1)_12_**	V	1	0.009	0.010	0.958	0.338	13.498
**ARIMAX(1,1,1)(2,1,1)_12_**	V	7	0.015	0.009	1.585	0.114	11.911**
**ARIMAX(1,1,1)(2,1,1)_12_**	V	9	-0.013	0.010	-1.368	0.172	12.520
**ARIMAX(1,1,1)(2,1,1)_12_**	W	5	-0.039	0.029	-1.318	0.188	12.690
**ARIMAX(1,1,1)(2,1,1)_12_**	W	7	-0.023	0.029	-0.790	0.430	13.786
**ARIMAX(1,1,1)(2,1,1)_12_**	W	8	0.043	0.029	1.476	0.141	12.241**
**ARIMAX(1,1,1)(2,1,1)_12_**	W	10	-0.045	0.029	-1.557	0.120	11.994**
**ARIMAX(1,1,1)(2,1,1)_12_**	T	2	0.018	0.009	2.082	0.038*	7.100**
	T	7	0.019	0.009	2.271	0.024*	
**ARIMAX(1,1,1)(2,1,1)_12_**	R	12	0.023	0.007	3.167	0.002*	1.470**
	T	7	0.021	0.009	2.482	0.013*	
**ARIMAX(1,1,1)(2,1,1)_12_**	R	12	0.022	0.007	3.083	0.002*	2.840**
	T	2	0.019	0.008	2.202	0.028*	
**ARIMAX(1,1,1)(2,1,1)_12_**	R	12	0.023	0.007	3.282	0.001*	-1.430**
	T	7	0.021	0.008	2.500	0.013*	
	T	2	0.019	0.008	2.214	0.027*	

T: temperature, R: rainfall, V: vapor pressure, W: wind speed

*: *p*-value<0.05

**: AIC value<12.408

### Forecasting BD cases

**Fig 4:**
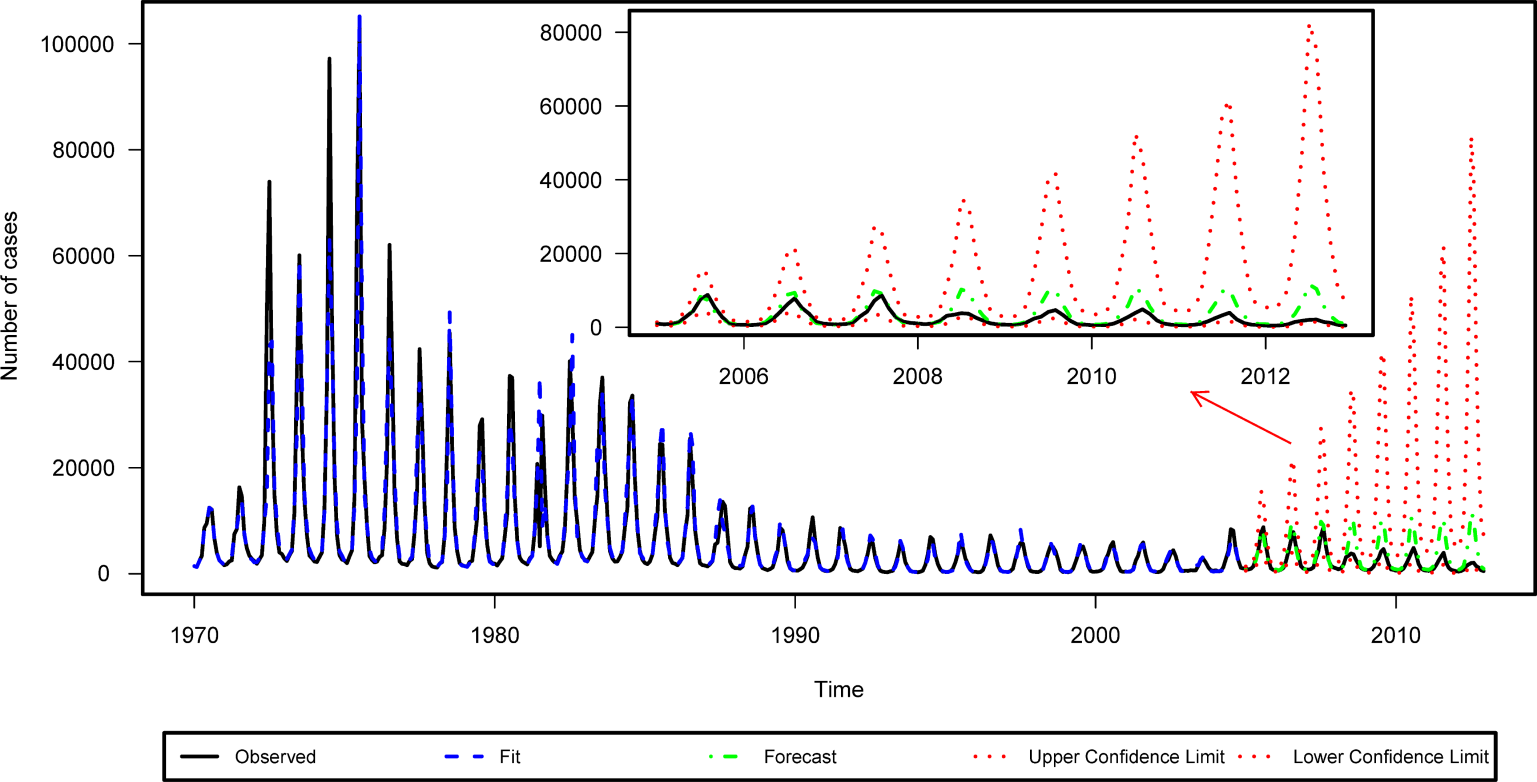
Results of prediction by the ARIMAX model with covariates of temperature at lag 7 and rainfall at lag 12.

This section attempted to predict monthly BD cases from January 2005 to December 2012 based on the ARIMAX models constructed above. The MSE of monthly BD cases predicted by different ARIMAX models were delineated in Table 4. Obviously, the ARIMAX model with covariates of temperature at lag 7 and rainfall at lag 12 had the lowest MSE, indicating the uttermost prediction accuracy (Fig 4). Actually a conspicuous amelioration of prediction accuracy was noticed: the MSE of the ARIMAX model with these two covariates (temperature at lag 7 and rainfall at lag 12) reduced by as high as 12% compared to the ARIMA model without them. But the ARIMAX model with covariates of temperature at lags 2, 7 and rainfall at lag 12 with the lowest AIC value failed to predict the most accurately. Obviously, temperature at lag 2 could not improve the prediction accuracy as the ARIMAX models actually predicted worse after covariate of temperature at lag 2 was added. Thus only temperature at lag 7 and rainfall at lag 12 could conspicuously improve the prediction accuracy. According to the ARIMAX model with the best predictive accuracy, temperature with 7-month lag (β = 0.021, 95%CI: 0.004–0.038) and rainfall with 12-month lag (β = 0.023, 95% CI: 0.009–0.037) could impact on the incidence of BD in Beijing. Yet despite the accurate prediction especially in the first three years, a decrease of accuracy was noticed as the forecast time gradually went onward. But generally speaking, the predictions were not bad as the observation values all fell within the 95% prediction limits (Fig 4). It is reasonable to say that the ARIMAX model was better at making short-term forecasts and entailed constant modification with newly available observations. Hence, the best model was updated and the monthly BD cases from January 2010 to December 2012 were re-predicted. The prediction accuracy was thus remarkably improved. (Results were laid out in S1 Fig).

**Table 4:** The MSE of prediction by different ARIMAX models from January 2005 to December 2012.

Model	Meteorological factors		MSE
	Variables	Lag	
**ARIMA(1,1,1)(2,1,1)_12_**	-	-	7328548.766
**ARIMAX(1,1,1)(2,1,1)_12_**	T	2	7674520.082
**ARIMAX(1,1,1)(2,1,1)_12_**	T	7	6562909.674
**ARIMAX(1,1,1)(2,1,1)_12_**	R	12	7250578.997
**ARIMAX(1,1,1)(2,1,1)_12_**	T	2	6920777.322
	T	7	
**ARIMAX(1,1,1)(2,1,1)_12_**	T	7	6448083.157
	R	12	
**ARIMAX(1,1,1)(2,1,1)_12_**	T	2	7558358.205
	R	12	
**ARIMAX(1,1,1)(2,1,1)_12_**	T	2	6768899.895
	T	7	
	R	12	

## Discussion

In our study, correlation analysis between BD cases and meteorological factors was conducted based on the prewhitened data by the ARIMA(1,1,1)(2,1,1)_12_ model. Except relative humidity, all the other factors, namely monthly temperature, rainfall, vapor pressure, and wind speed at different lags, were all found to be associated with the number of monthly BD cases, but only temperature and rainfall could eventually be incorporated into the ARIMAX models. In comparison, a study in Changsha reported that monthly temperature, relative humidity, air pressure, maximum temperature, and minimum temperature were significantly correlated with BD counts[14]. Another study in Jinan found that maximum temperature, minimum temperature, rainfall, relative humidity, and air pressure were all significantly correlated with the number of BD[12]. Additionally, according to a study set in Beijing with data on a daily basis, mean temperature, relative humidity, rainfall, wind speed and duration of sunshine all played roles in BD transmission [22]. Conclusions varied among a whole range of local climate conditions, different data scales and analysis methods.

The ARIMAX model showed that only temperature at lags 2, 7 and rainfall at lag 12 could improve goodness of fit. As for the AIC value of ARIMAX model with single lagged meteorological factor, meteorological factors in order of importance were rainfall (at lag 12), temperature (at lag 7) and temperature (at lag 2). In contrast, previous studies almost took temperature as the most important factor[10, 12, 14, 16]. Furthermore, the impact of rainfall on BD in the past research was inconsistent, varying from positive to negative association and even no relationship at all. A previous study in Beijing found a positive association between BD cases and rainfall[22], and similar findings have also been reported in Taiwan[23]. Yet negative association between BD cases and rainfall was also documented by studies in Wuhan[15] and in Sub-Saharan Africa[24]. Unlike these studies, some Changsha researchers concluded that there was no relationship between BD cases and rainfall[14]. The discrepancy might arise from the distinction in local climate conditions. In our study, four ARIMAX models with multiple variables could lower the AIC value. The one with the lowest AIC value was the ARIMAX model with covariates of temperature at lags 2, 7 and rainfall at lag 12 because it contained all meteorological factors with an impact on BD.

Predictive accuracy of different ARIMAX models was evaluated by MSE. However, it was the ARIMAX model with covariates of temperature at lag 7 and rainfall at lag 12 that predicted the most accurately rather than the ARIMAX model with the best goodness of fit. For single meteorological factor, both temperature at lag 7 and rainfall at lag 12 could obviously improve the predictive accuracy, but prediction made by temperature at lag 2 was even worse than the ARIMA model without any covariate. For the ARIMAX models with multiple independent variables, the prediction accuracy was lower than those with removal of covariate of temperature at lag 2. Temperature at lag 2 was the least important of the three meteorological factors. Therefore, important meteorological factors might ameliorate prediction accuracy. Another conclusion reached by this research was that the ARIMAX model was more adept at short-term predictions, as the predictive accuracy decreased gradually as the forecast time went onward. Such a result would suggest the latest available data to be incorporated into the updated model, which turned out to be a simpler means of updating.

The long-term effect of meteorological factors on BD cases indicated by this study also deserves attention. Our study demonstrated the obvious presence of an impact of 7-month lagged temperature and 12-month lagged rainfall on BD incidence in contrast with an average of 3–4 weeks lag suggested by most previous studies[11, 16, 25]. Yet our study was not alone in the field, take for instance a study on leptospirosis transmission in Thailand[26]. Equally adopting the ARIMAX model, the researchers discovered that the model with covariate of 8-months lagged rain was the best model for the northern region, while the model with covariates of 10-months lagged rain and 8-months lagged temperature was the best for the northeastern region.

Yet this study is not free from limitations. Our analysis was conducted on ground of monthly data while weekly data or daily data could probably improve the accuracy of lagged time estimate. Due to the imperfect disease surveillance system, however, only monthly surveillance data were available to this study, which entails further improvement in the notification and dissemination of health-related data in a well-established surveillance system. In addition, the sources of data were collected from one single study area Beijing with limited sample size. Thus the result might be subject to influence from local climate conditions, populations, and ecological characteristics. Comparisons among different places were therefore necessary to attain results free from confounding factors, which hopefully can be fulfilled in future studies. Apart from the data collection, appropriate interpretation of the results also remained to be a problem. Results such as the long term effect of meteorological factors on dysentery were calculated purely by rigorous mathematical methods while the biological mechanism behind was still far from clear. Hopefully future studies might reveal the mechanism behind such a delay of impact. Additionally, with focus on the relationship between meteorological variables and BD cases, the role other relevant factors might play in BD incidence went largely unnoticed such as economic development, improvement in medical care, population growth. Another problem was the slight changes to the diagnosis of BD over the long study period. Although no marked changes were observed and the few changes were almost limited to classification of the BD subtypes, there might still be influence on the final outcomes to a certain extent. Considering the overall continuity exhibited by authoritative official statistics, we suppose the association would not be significantly affected.

In conclusion, this study demonstrated a successful application of the ARIMAX model to the time series data on BD cases in Beijing. The effectiveness of such a model was evident from an accurate goodness of fit and high predictive accuracy especially in the short term. In this sense, health sectors, practitioners and other authorities concerned might benefit from this research and take full consideration of meteorological factors as well as the lag effects with a view to rationally disposing health resources, intervening in possible BD epidemics, and forging healthcare strategies accordingly. With this study, earlier public health measures can be taken because even without enough data on meteorological factors, the ARIMA model could still predict BD prevalence in the next two or three years accurately. When data on climatic variables are available, the prediction accuracy could be further ameliorated through the ARIMAX model.

## Supporting information

S1 Fig Results of prediction by the updated ARIMAX model. The parameters of the ARIMAX(1,1,1)(2,1,1)_12_ model with covariates of temperature at lag 7 and rainfall at lag 12 were updated by fitting the monthly BD cases and weather factors data from January 1970 to December 2009, while the model structure remained unchanged. A new prediction was made by the updated ARIMAX model from January 2010 to December 2012. Obviously, the new forecast was more accurate than the previous one.
(TIFF)

S1 File Details for ARIMA model analysis. (DOCX)

S1 Table The monthly data of bacillary dysentery cases and meteorological factors in Beijing from 1970 to 2012. (XLSX)
