# Supplementary figures and images for: Impact of meteorological factors on the incidence of bacillary dysentery in Beijing, China: A time series analysis (1970-2012)
Source: PLoS One. 2017 Aug 10;12(8):e0182937. doi: 10.1371/journal.pone.0182937 (PMC5552134; doi:10.1371/journal.pone.0182937)

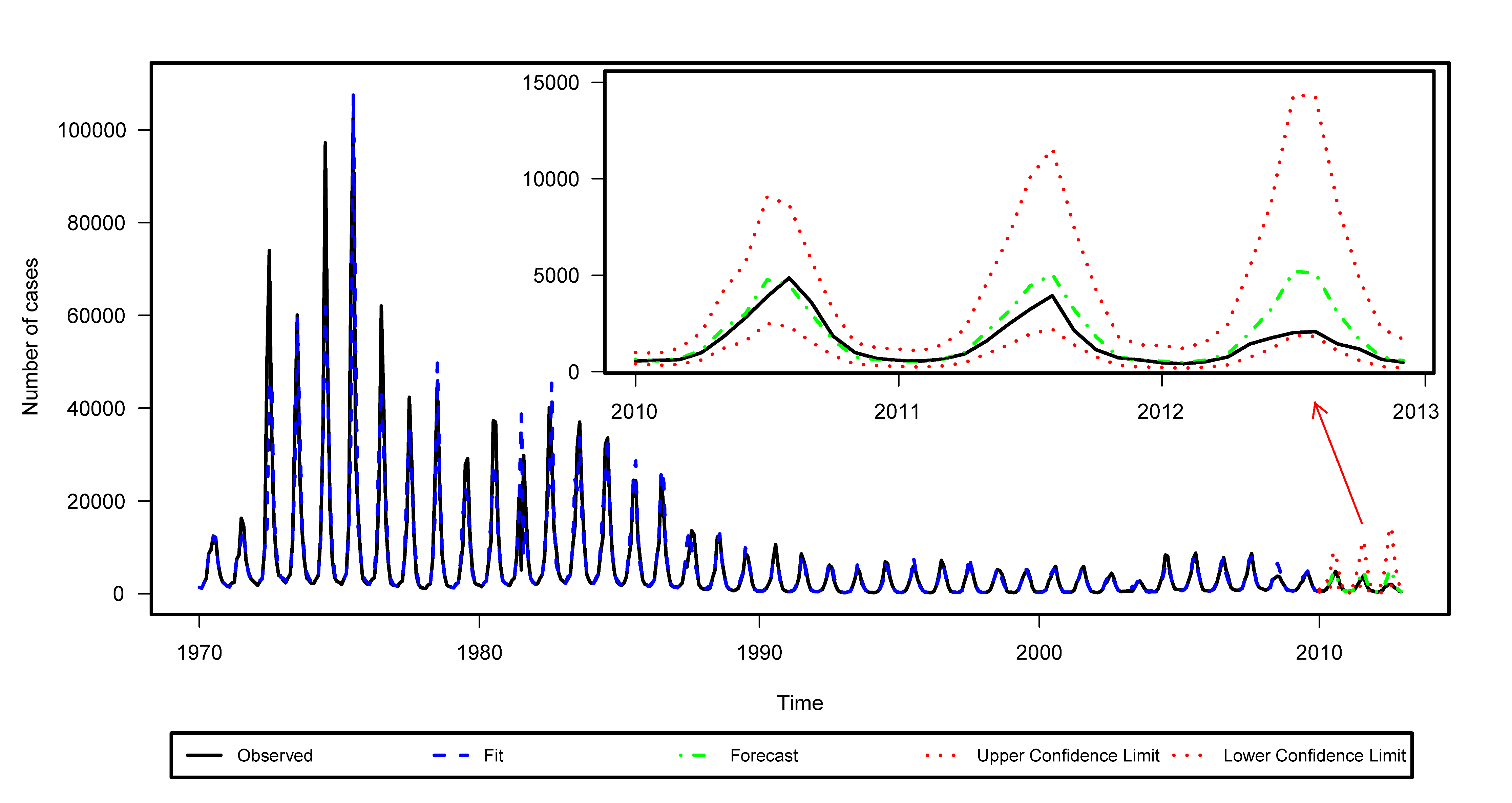

Supplement: S1 Fig — The parameters of the ARIMAX(1,1,1)(2,1,1)12 model with covariates of temperature at lag 7 and rainfall at lag 12 were updated by fitting the monthly BD cases and weather factors data from January 1970 to December 2009, while the model structure remained unchanged. A new prediction was made by the updated ARIMAX model from January 2010 to December 2012. Obviously, the new forecast was more accurate than the previous one. (TIFF) [file pone.0182937.s001.tiff]
