# Supplementary material for: Impact of meteorological factors on the incidence of bacillary dysentery in Beijing, China: A time series analysis (1970-2012)
Source: PLoS One. 2017 Aug 10;12(8):e0182937. doi: 10.1371/journal.pone.0182937 (PMC5552134; doi:10.1371/journal.pone.0182937)
Supplement: S1 File — (DOCX) [file pone.0182937.s002.docx]

**Details for the analysis of the ARIMA model**

BD cases series from 1970 to 2004 were plotted in Fig 1. As was shown in Fig 1, the series displayed considerable variation and obvious seasonal distribution, so a nonstationary seasonal ARIMA model seemed to be reasonable. Obviously, the BD cases series fluctuated within a large range. To stabilize the fluctuation, logarithmic transformation was performed and Consequently the series behaved much more stationary after transformation (Fig 2).


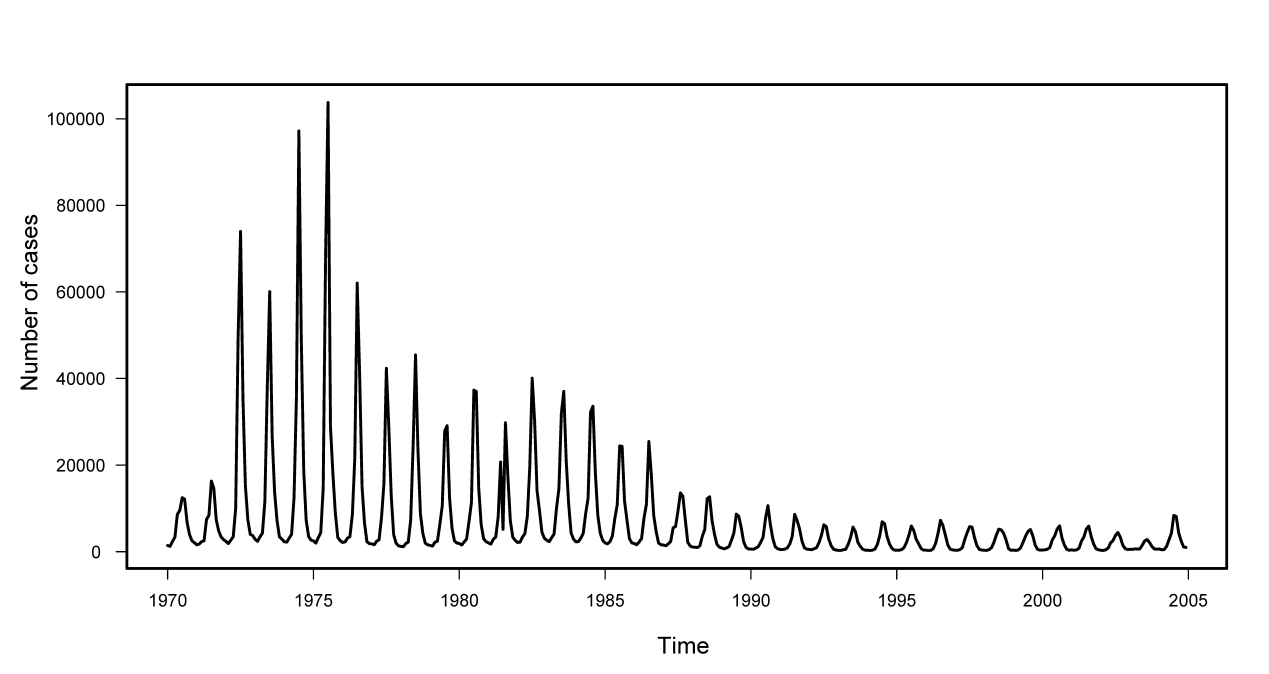


**Fig 1. The monthly number of BD cases in Beijing, 1970-2004.**


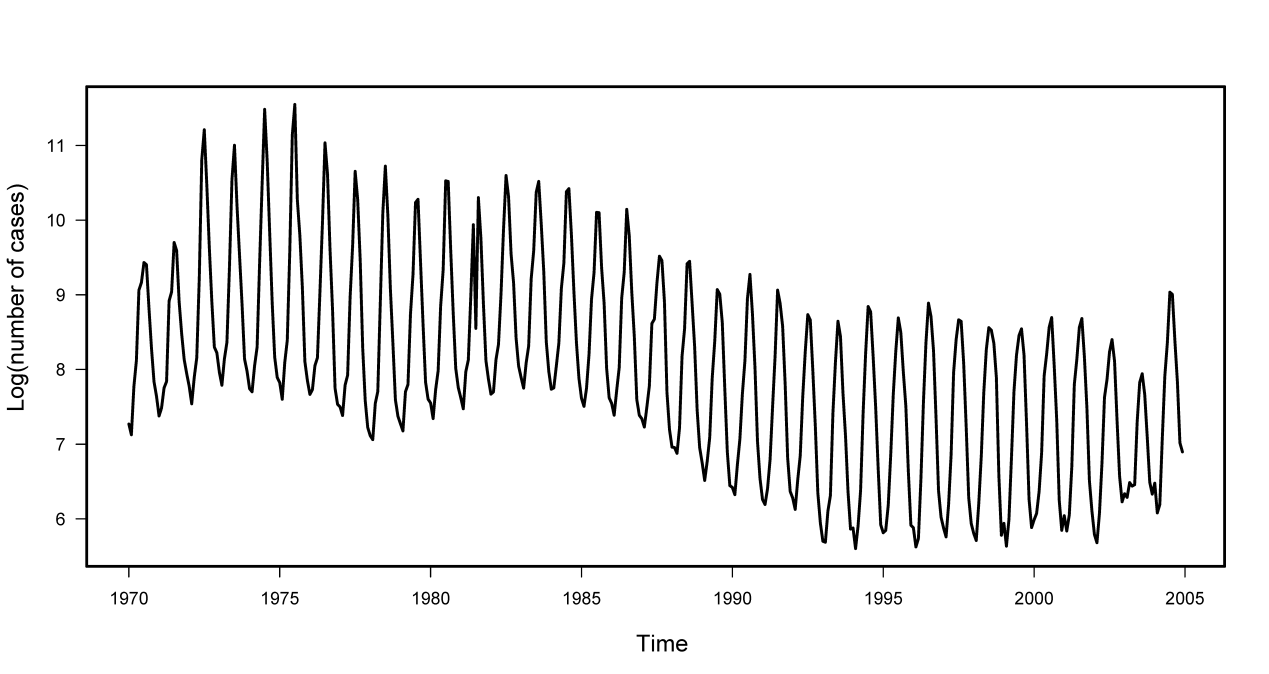


**Fig 2. The BD cases series after logarithmic transformation.**

After logarithmic transformation, the series plot presented a downward trend along with an obvious seasonal distribution, so a first order non-seasonal difference was applied in the first step (Fig 3). Then secondly the ACF and PACF graphs were also plotted (Fig 4).


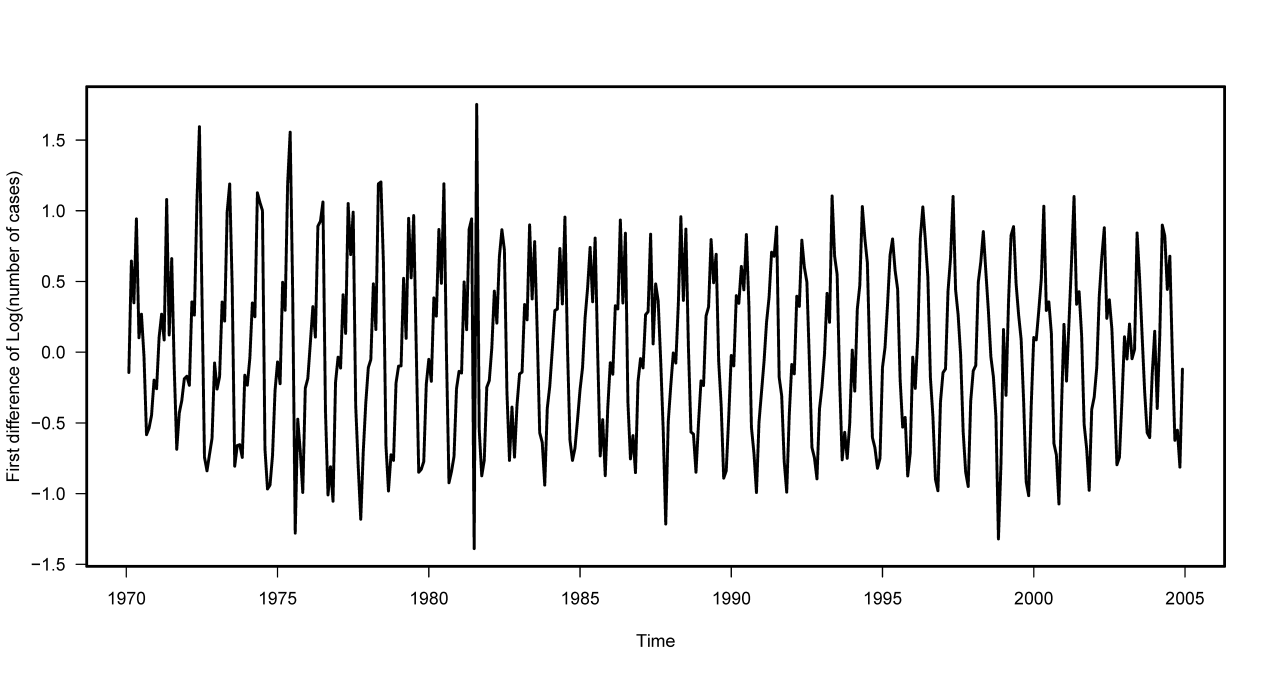


**Fig 3. Time series plot of the first difference of logarithms for BD cases.**


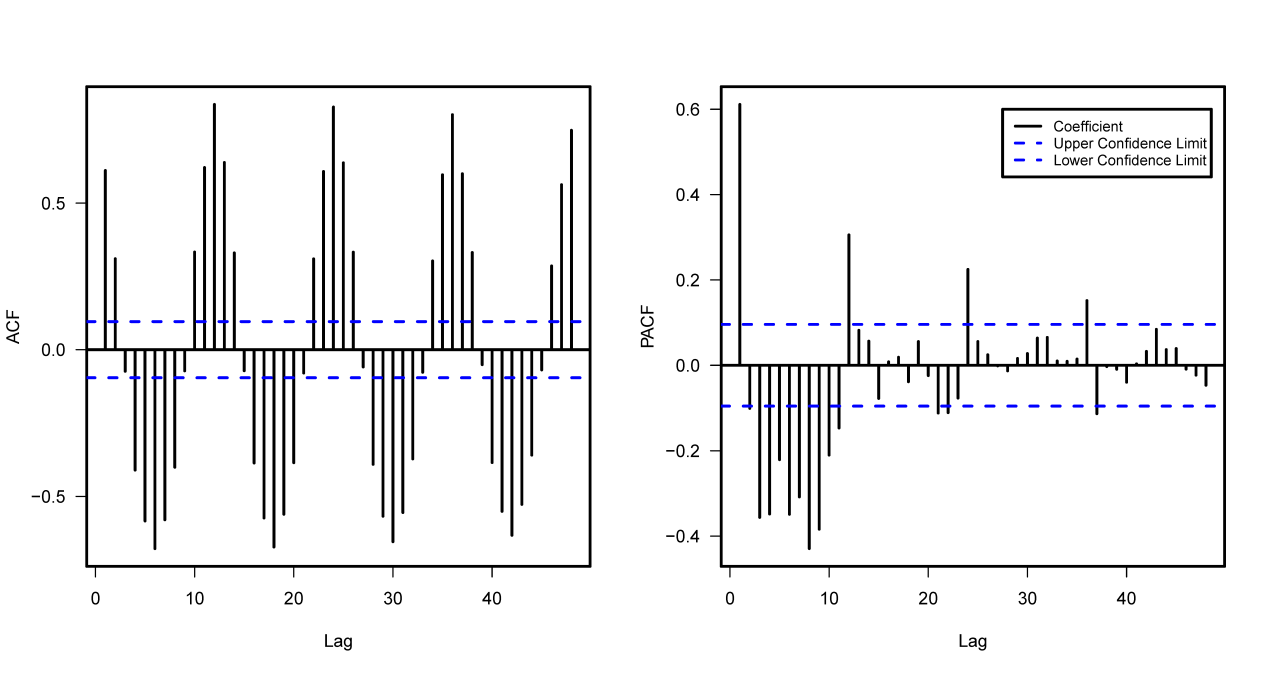


**Fig 4. The ACF and PACF plots of the first difference of logarithms for BD cases.**

The general downward trend disappeared but the strong seasonality was not absent, as can be seen by the behavior shown in Fig 4. In order to acquire a stationary series, seasonal differencing was carried out. Delineated in Fig 5 was the time series plot of the BD cases after the performance of both first order and seasonal differences. Obviously, most of the seasonality disappeared this time. Fig 6 further demonstrated that very little autocorrelation remained in the series after carrying out these two differences.


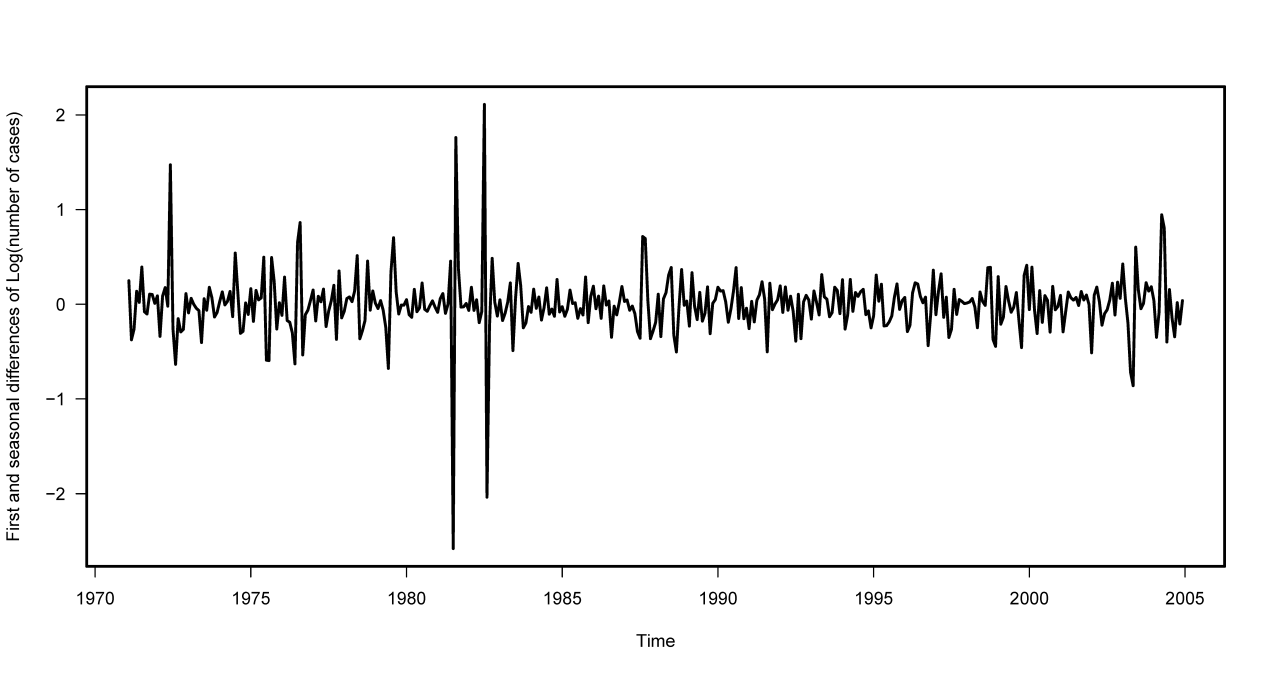


**Fig 5. Time series plot of the first and seasonal differences of logarithms for BD cases.**


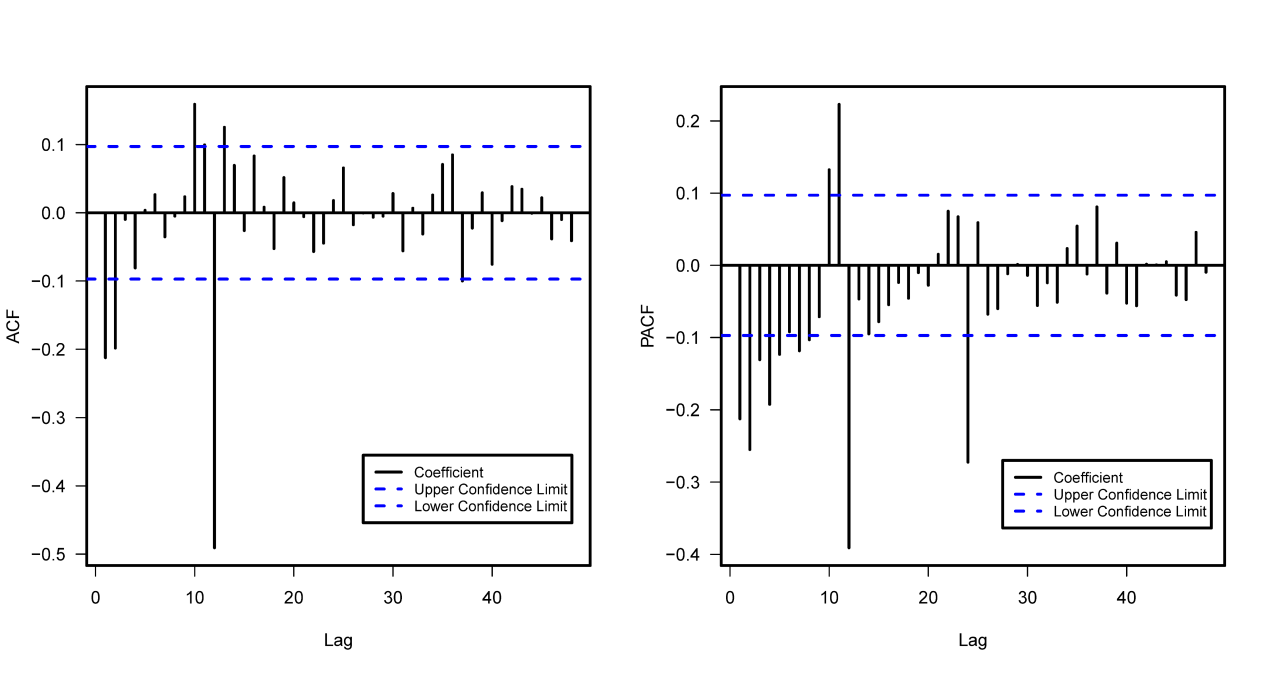


**Fig 6. ACF and PACF plots of the first and seasonal differences of logarithms for BD cases.**

ACF combined with PACF plots (ACF&PACF plots) acted as good guidelines to identify the model structure. Summarized in Table 1 was the relationship between ARMA models and the behavior of the ACF&PACF plots. For pure AR(*p*) or MA(*q*) models, and even for the mixed ARMA(*p,q*) model, the parameter *q* did not exceed the maximum lag cutting off in ACF, and neither did the parameter *p* exceed the maximum lag cutting off in PACF. Notice that the sample ACF values exceeded the critical value at lags 1, 2, 10, 12 and 13, the maximum value of the seasonal parameter *Q* and that of the non-seasonal parameter *q* was 1 and 2 respectively. Lags 10 and 13, also neighbors of seasonal lag 12, arose from the multiplication of seasonal and non-seasonal factors. Similarly, because the sample PACF values were significant at lags 1, 2, 3, 4, 5, 7, 8, 10, 11, 12 and 24，the maximum value of the seasonal parameter *P* and that of the non-seasonal parameter *p* should be 2 and 8 respectively. Lags 10 and 11 might be resulted from the multiplication of seasonal and non-seasonal factors. Considering the non-seasonal part of ACF cuts off after lag 2, an ARMA(0,2) model might be reasonable for describing the non-seasonal part of the BD cases series. Hence the AR order *p* could be reduced to a small value or even to zero. For simplicity, the maximum value of *p* was assumed to be 2. In order to find the most suitable model, we searched all 54 ARIMA models under the following conditions: $p=0, 1 or 2;q=0, 1 or 2;P=0, 1 or 2;Q=0 or 1$. Results were summarized in Table 2. Then we analyzed the five ARIMA models with the lowest AIC value, among which the ARIMA(1,1,1)(2,1,1)_12_ model was the most suitable one. We also analyzed ARIMA models with increased AR order such as ARIMA(3,1,1)(2,1,1)_12_, ARIMA(4,1,1)(2,1,1)_12_ and ARIMA(5,1,1)(2,1,1)_12_, but the estimations of autoregressive coefficients with the order larger than 1 were not statistically significant. In this sense, complex models with non-seasonal AR orders above 1 are unnecessary and the ARIMA(1,1,1)(2,1,1)_12_ model was by far the most suitable one.

**Table 1. General behavior of the ACF and PACF for ARMA models.**

|  | AR(*p*) | MA(*q*) | ARMA(*p*,*q*),*p*>0,*q*>0 |
| --- | --- | --- | --- |
| ACF | Tails off | Cuts off after lag *q* | Tails off |
| PACF | Cuts off after lag *p* | Tails off | Tails off |

**Table 2. Results of all 54 ARIMA models.**

| ARIMA(*p*,*d*,*q*)(*P*,*D*,*Q*)_12_ | | | | | | AIC | Ljung-Box test | | |
| --- | --- | --- | --- | --- | --- | --- | --- | --- | --- |
| *P* | *d* | *q* | *P* | *D* | *Q* |  | Statistic | DF | significance |
| 1 | 1 | 2 | 2 | 1 | 1 | 11.086 | 6.079 | 6 | 0.414 |
| 2 | 1 | 1 | 2 | 1 | 1 | 11.155 | 6.287 | 6 | 0.392 |
| 1 | 1 | 1 | 2 | 1 | 1 | 12.408 | 8.758 | 7 | 0.271 |
| 2 | 1 | 2 | 2 | 1 | 1 | 12.761 | 5.518 | 5 | 0.356 |
| 2 | 1 | 2 | 1 | 1 | 1 | 14.834 | 5.712 | 6 | 0.456 |
| 2 | 1 | 1 | 0 | 1 | 1 | 15.970 | 8.652 | 8 | 0.372 |
| 1 | 1 | 2 | 0 | 1 | 1 | 15.979 | 8.508 | 8 | 0.386 |
| 0 | 1 | 2 | 2 | 1 | 1 | 16.331 | 14.869 | 7 | 0.038 |
| 2 | 1 | 1 | 1 | 1 | 1 | 16.507 | 8.802 | 7 | 0.267 |
| 1 | 1 | 2 | 1 | 1 | 1 | 16.514 | 8.687 | 7 | 0.276 |
| 1 | 1 | 1 | 0 | 1 | 1 | 17.568 | 10.772 | 9 | 0.292 |
| 2 | 1 | 2 | 0 | 1 | 1 | 17.697 | 7.860 | 7 | 0.345 |
| 1 | 1 | 1 | 1 | 1 | 1 | 18.777 | 11.727 | 8 | 0.164 |
| 0 | 1 | 2 | 0 | 1 | 1 | 22.197 | 20.379 | 9 | 0.016 |
| 0 | 1 | 2 | 1 | 1 | 1 | 22.596 | 20.401 | 8 | 0.009 |
| 1 | 1 | 1 | 2 | 1 | 0 | 22.799 | 7.740 | 8 | 0.459 |
| 1 | 1 | 2 | 2 | 1 | 0 | 22.928 | 6.155 | 7 | 0.522 |
| 2 | 1 | 1 | 2 | 1 | 0 | 23.060 | 6.360 | 7 | 0.498 |
| 2 | 1 | 2 | 2 | 1 | 0 | 24.298 | 5.291 | 6 | 0.507 |
| 0 | 1 | 2 | 2 | 1 | 0 | 26.806 | 13.458 | 8 | 0.097 |
| 2 | 1 | 0 | 2 | 1 | 1 | 48.799 | 27.557 | 7 | 0.000 |
| 2 | 1 | 2 | 1 | 1 | 0 | 49.801 | 12.937 | 7 | 0.074 |
| 2 | 1 | 0 | 0 | 1 | 1 | 53.004 | 28.852 | 9 | 0.001 |
| 2 | 1 | 0 | 1 | 1 | 1 | 54.424 | 29.597 | 8 | 0.000 |
| 1 | 1 | 1 | 1 | 1 | 0 | 55.749 | 14.419 | 9 | 0.108 |
| 1 | 1 | 2 | 1 | 1 | 0 | 56.881 | 12.961 | 8 | 0.113 |
| 2 | 1 | 1 | 1 | 1 | 0 | 56.990 | 13.212 | 8 | 0.105 |
| 2 | 1 | 0 | 2 | 1 | 0 | 57.926 | 26.071 | 8 | 0.001 |
| 0 | 1 | 2 | 1 | 1 | 0 | 61.218 | 16.988 | 9 | 0.049 |
| 0 | 1 | 1 | 0 | 1 | 1 | 61.648 | 40.262 | 10 | 0.000 |
| 0 | 1 | 1 | 2 | 1 | 0 | 63.156 | 39.125 | 9 | 0.000 |
| 0 | 1 | 1 | 1 | 1 | 1 | 63.639 | 40.421 | 9 | 0.000 |
| 0 | 1 | 1 | 2 | 1 | 1 | 64.624 | 39.274 | 8 | 0.000 |
| 1 | 1 | 0 | 2 | 1 | 1 | 66.545 | 34.365 | 8 | 0.000 |
| 1 | 1 | 0 | 0 | 1 | 1 | 69.561 | 33.566 | 10 | 0.000 |
| 1 | 1 | 0 | 1 | 1 | 1 | 71.315 | 34.100 | 9 | 0.000 |
| 0 | 1 | 0 | 2 | 1 | 1 | 74.393 | 31.535 | 9 | 0.000 |
| 0 | 1 | 0 | 0 | 1 | 1 | 76.136 | 30.560 | 11 | 0.001 |
| 1 | 1 | 0 | 2 | 1 | 0 | 76.614 | 34.391 | 9 | 0.000 |
| 0 | 1 | 0 | 1 | 1 | 1 | 77.581 | 31.030 | 10 | 0.001 |
| 0 | 1 | 0 | 2 | 1 | 0 | 89.722 | 35.224 | 10 | 0.000 |
| 2 | 1 | 0 | 1 | 1 | 0 | 93.529 | 35.125 | 9 | 0.000 |
| 0 | 1 | 1 | 1 | 1 | 0 | 96.317 | 48.500 | 10 | 0.000 |
| 1 | 1 | 0 | 1 | 1 | 0 | 112.759 | 47.175 | 10 | 0.000 |
| 0 | 1 | 0 | 1 | 1 | 0 | 128.720 | 48.821 | 11 | 0.000 |
| 2 | 1 | 2 | 0 | 1 | 0 | 141.206 | 82.734 | 8 | 0.000 |
| 1 | 1 | 1 | 0 | 1 | 0 | 156.113 | 94.602 | 10 | 0.000 |
| 1 | 1 | 2 | 0 | 1 | 0 | 157.570 | 92.151 | 9 | 0.000 |
| 2 | 1 | 1 | 0 | 1 | 0 | 157.736 | 92.910 | 9 | 0.000 |
| 0 | 1 | 2 | 0 | 1 | 0 | 162.144 | 91.966 | 10 | 0.000 |
| 2 | 1 | 0 | 0 | 1 | 0 | 201.281 | 132.119 | 10 | 0.000 |
| 0 | 1 | 1 | 0 | 1 | 0 | 203.693 | 150.211 | 11 | 0.000 |
| 1 | 1 | 0 | 0 | 1 | 0 | 226.525 | 155.585 | 11 | 0.000 |
| 0 | 1 | 0 | 0 | 1 | 0 | 243.290 | 154.694 | 12 | 0.000 |
